# Management of Vertebral Fragility Fracture in Older People: Recommendations from a Spanish Consensus of Experts

**DOI:** 10.3390/geriatrics9020024

**Published:** 2024-02-23

**Authors:** Santos Castañeda, Carmen Navarro Ceballos, Jaqueline Usón Jaeger, Carolina de Miguel Benadiba, Esteban Gómez Martín, Guillermo Martínez Díaz-Guerra, Luis Alvarez-Galovich

**Affiliations:** 1 Rheumatology Service, Hospital Universitario La Princesa, IIS-Princesa, 28006 Madrid, Spain; 2 Cátedra UAM-Roche, EPID-Future, Department of Medicine, Autonomous University of Madrid (UAM), 28049 Madrid, Spain; 3 Geriatric Service, Hospital Universitario Severo Ochoa, Leganés, 28914 Madrid, Spain; cnavarro13313@gmail.com; 4 Rheumatology Service, Hospital General Universitario de Móstoles, Móstoles, 28935 Madrid, Spain; jusonjaeger@gmail.com; 5 Rehabilitation Service, Hospital Universitario Ramón y Cajal, 28034 Madrid, Spain; cdemibe@gmail.com; 6 Primary Care Center Zulema, Alcalá de Henares, 28810 Madrid, Spain; gomezmartinesteban@hotmail.com; 7 Endocrinology Service, Hospital Universitario 12 de Octubre, 28041 Madrid, Spain; guillermo.martinez@salud.madrid.org; 8 Spine Unit, Hospital Universitario Fundación Jiménez Díaz, 28040 Madrid, Spain; lalvarez@fjd.es

**Keywords:** osteoporosis, vertebral fractures, older patients, aging, comprehensive assessment, expert consensus

## Abstract

Vertebral fragility fractures (VFF) pose a challenge for appropriate care. The aim of this study was to develop consensus recommendations for the management of VFF in older people from a multidisciplinary approach. Specialists in osteoporosis belonging to different scientific societies reviewed the main clinical practice guidelines published in Spain in 2014. Thirty-five recommendations for the management of VFF were evaluated by seven experts using an anonymous survey. Consensus was defined as 80% of responses of 8 (agree) and 9 (strongly agree) on a Likert scale. Consensus was achieved in 22 recommendations (62.8%). The experts agreed on the need for anamnesis, clinical assessment, and laboratory tests, including erythrocyte sedimentation rate, proteinography, and the assessment of levels of calcium, vitamin D, alkaline phosphatase, and thyroid-stimulating hormone. Optional tests, such as bone turnover markers (BTMs), magnetic resonance imaging, bone scintigraphy, or using a fracture risk assessment tool (FRAX^®^), did not achieve an agreed consensus. Also, there was consensus regarding the administration of calcium/vitamin D supplements, the withdrawal of toxic habits, and personalized physical exercise. Participants agreed on the administration of teriparatide for 24 months and then a switch to denosumab or bisphosphonates in patients at high risk of fracture. Specialists in osteoporosis, primary care physicians, and geriatricians should be involved in the follow-up of patients with VFF. Although there was multidisciplinary agreement on diagnostic tests and non-pharmacological and pharmacological treatment in frail older people, therapeutic objectives should be individualized for every patient. In addition to the specific recommendations, close collaboration between the geriatrician and the primary care physician is essential for the optimal chronic management of frail patients with fragility fractures.

## 1. Introduction

Enhanced bone fragility and increased risk of fractures due to low bone mass and the deterioration of the bone tissue microarchitecture are the main features of osteoporosis [1]. Older subjects with osteoporosis are at high or very high risk of fragility or low-impact fractures, along with an increase in morbidity and mortality [2]. Osteoporosis is also a complex disease that involves the participation of many specialists according to the different clinical expressions and phenotypic profiles of older people [3,4].

Among osteoporotic (fragility) fractures, vertebral fractures are the most frequent. Otherwise, frailty, defined as a state with decreased physiological reserve and increased vulnerability to stress, is identified in about 70% of patients admitted to a hospital for vertebral fragility fracture (VFF) using recognized clinical scales for frailty assessment [5]. Overall, frail patients are more vulnerable and are at high risk of falls. Moreover, subjects who suffer from VFF are more likely to suffer further falls, with a fear of falling, depression and anxiety symptoms, impairment of social function, reduced physical activity, and negative impacts on their health-related quality of life (HRQoL) [6]. Data from a systematic review based on 19 studies of patients with VFF showed an incidence of hospitalization ranging between 2.8 and 19.3 per 10,000 patients/year, a rate of in-hospital mortality between 0.9% and 3.5%, and a rate of discharge to a healthcare facility between 34% and 50% [7]. In addition, higher mortality and a prolonged length of hospital stay were associated with increasing comorbidities and older age [7]. Accurate diagnosis is not established in as many as two-thirds of patients with VFF that occur each year, and consequently, these patients are not treated, although, in subjects at high risk of fragility fracture, the implementation of screening procedures and adequate treatment are cost-effective [8]. Sarcopenia screening is also very important due to the increasing risk of falls after 65–70 years, particularly in the oldest population (age ≥ 80 years), and the well-known physical, social, and psychological consequences. Also, the effect of VFF on quality of life is greater and is of longer duration compared with the impacts of hip fractures and other types of osteoporotic fractures [9]. Furthermore, due to the global pattern of population aging, an increase in the overall burden of osteoporosis and VFF may be expected in the forthcoming years.

**Figure 1:**
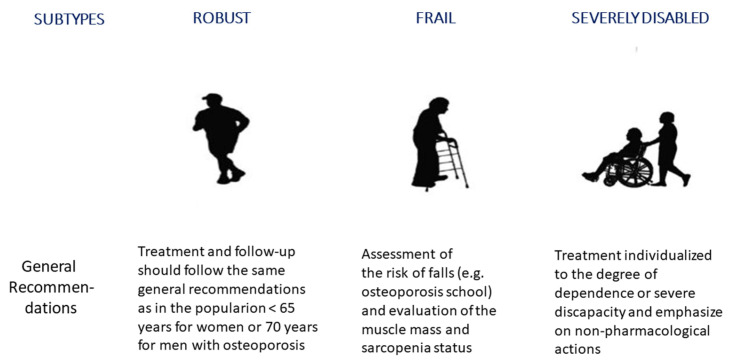
Classification of older people subjects in functional subtypes—general recommendations for the treatment and follow-up of vertebral fragility fractures. The conventional definition of “older people” as a chronological age of 65 or older is currently outdated due to a global improvement in life conditions and life expectancy of the population in the last decades, especially in developed countries. Cartoons modified from: https://geri-arte.com/el-continuo-funcional-del-anciano-robusto-a-la-dependencia-grave/ (accessed on 23 October 2023).

In daily practice, however, the challenges faced by specialists are multifaceted strategies for appropriate risk assessment, diagnostic approaches, non-pharmacological management, and the pharmacological treatment of osteoporosis and VFF in older people [10,11]. Moreover, the use of the chronological age of 65 or older for defining “older people” is currently outdated due to a global improvement in the life conditions, diet, and life expectancy of the population in recent decades, especially in developed countries. Therefore, this definition may no longer be adequate for an increased healthy life expectancy of the population, rather than simply the time spent alive [12]. Indeed, older people encompass a very heterogeneous age range and life qualities. Older people experience a transition from a healthy, robust state with good physical, mental, and social functioning to a fragility state. Moreover, frailty is a dynamic concept that can worsen through the accumulation of deficits and finally end in disability, also known as severe frailty. Furthermore, frailty can be influenced by many factors like comorbidity, genetic and environmental factors, and the presence of geriatric syndromes. Based on global trends in aging, we are able to classify older people into three subtypes: robust, frail, and severely disabled or dependent subjects [13,14,15] (Figure 1).

In addition, there is a wide range of criteria within the clinical practice guidelines for the treatment of osteoporosis under the perspective of different medical specialties [16,17,18,19,20,21,22,23,24], with limited and inconsistent recommendations for the integral care of older people with VFF.

Therefore, a project based on a consensus of experts was designed to integrate guidelines of different Spanish scientific societies in order to develop some recommendations for the management and follow-up of VFF in older people from a multidisciplinary approach.

## 2. Materials and Methods

### 2.1. Study Design

The study project was conducted between June and September 2021, being the primary objective to reconcile the main guidelines on osteoporosis currently available in Spain in order to develop uniform recommendations for the treatment and follow-up of older people with VFF. The final purpose was to optimize the care of these old or very old patients from a multidisciplinary approach, considering the different possible clinical profiles. Approval of the study protocol by a clinical research ethics committee was not required because of the qualitative research nature of this study.

### 2.2. Participants and Questionnaire

Seven clinical experts (three women and four men) with at least 20 years of experience in osteoporosis and fragility fractures (one geriatrician, two rheumatologists, one endocrinologist, a family physician, one rehabilitation specialist, and one orthopedic surgeon) and one methodologist and other one documentalist formed the task group. Clinical experts from these disciplines were selected because these specialties are those most frequently involved in the care and long-term follow-up of patients with VFF in our healthcare setting.

The first step consisted of the selection and review of the main clinical practice guidelines on osteoporosis published by five different national scientific societies from 2014 to 2022, including the Spanish Society for Bone Research and Mineral Metabolism (SEIOMM) 2021 [25], the Spanish Society of General and Family Physicians (SEMG) 2015 [26], the Spanish Society of Traumatology and Orthopaedic Surgery (SECOT) 2015 [27], the Spanish Society of Family and Community Medicine (semFYC) 2014 [28], and the Spanish Society of Rheumatology (SER) 2018 [29]. In addition, the recommendations for the assessment and treatment of primary osteoporosis in women in the community of Madrid (CAM 2015) [30] and the consensus document on osteoporosis in males, 2018, by members of the Bone Metabolism Working Group of the Spanish Society of Endocrinology [31], were also reviewed. The review was focused on aspects related to fragility fractures in older people. The following steps were as follows: drafting of the different items applicable to older people with VFF; a meeting of the members of the expert committee to discuss and modify the content of the questionnaire; and a final process of development and validation of the definitive questionnaire by all members of the scientific committee. The questions were generated based on the information provided by the abovementioned guidelines.

The final questionnaire included 35 items grouped into four sections: diagnostic tests (7 items), non-pharmacological treatment (5 items), pharmacological treatment (5 items), and follow-up (18 items). Each question was formulated so that it could be answered using a 9-point Likert scale, from 1 = strongly disagree, 2 = disagree, 3 = moderately disagree, 4 = slightly disagree, 5 = neither agree nor disagree, 6 = slightly agree, 7 = moderately agree, 8 = agree, and 9 = strongly agree. The questionnaire was lodged on an internet microsite that participants accessed via a web link. The questionnaire was completed anonymously by the seven clinical experts of the scientific committee. Consensus in favor of the question/recommendation was defined when the sum of responses “agree” or “strongly agree” was equal to or greater than 80% of the total responses obtained for every specific item. Details of the study questionnaire are shown in the Supplementary Materials.

A double-entry verification of the answers obtained by all participants was carried out with a logical function. The Likert points obtained for the responses to each item are expressed as mean and standard deviation (SD).

## 3. Results

A total of 35 questions regarding the diagnosis, management, and follow-up of older patients with VFF were reviewed, and consensus was achieved in 22 items (62.8%).

In the first section of the diagnostic tests (Table 1), consensus was obtained in four of the seven items reported (57.1) regarding anamnesis and clinical assessment (100% agreement). In the additional item of the Fracture Risk Assessment Tool (FRAX^®^) score, consensus was not achieved. Also, all participants agreed on the need for laboratory tests, including serum calcium and vitamin D levels, the erythrocyte sedimentation rate, alkaline phosphatase (ALP), thyroid-stimulating hormone (TSH), protein electrophoresis, and a measurement of renal function. The need to perform dorsal and lumbar X-rays reached an agreement of 85.7%. There was no consensus on the requirement of other laboratory tests, such as immunofixation, bone turnover markers, and magnetic resonance imaging (MRI)/bone scintigraphy.

In the second section of non-pharmacological measures (Table 2), consensus was obtained in three of the five items (60%) analyzed, which included the recommendation of calcium/vitamin D intake, breaking toxic habits, and personalized physical exercise. Treatment with orthosis and a reduction in caffeine consumption were not considered relevant. In the third section of the questionnaire related to pharmacological treatment (Table 2), a consensus was achieved in only one of the five items evaluated (20%), which was the use of teriparatide for 24 months followed by denosumab or bisphosphonates.

Pharmacological treatment should be individualized to each patient, clinical scenario, and socioeconomic conditions of each country. In summary, bone-forming agents should be the first choice in patients with severe osteoporosis, followed by an antiresorptive agent. Renal function, oral tolerance to medication, and comorbidities should also be considered. In severe cases, and exceptionally, a combined treatment with two agents with different mechanisms of action (e.g., a bone-forming agent and an antiresorptive drug) for a limited period of time may be indicated.

In the final section of the follow-up of older people with VFF, a consensus was reached in 14 of the 18 items (77.8%) (Table 3). In this regard, all experts agreed with the actions to be implemented depending on the classification of the subtypes of older people into robust (85.7% agreement), frail (100%), and severely disabled subjects (85.7%). They also agreed that robust older people should be followed by primary care physicians (100%) and frail or severely disabled patients by geriatricians (85.7%). There was 100% agreement on the fact that primary care physicians and/or specialists in metabolic bone diseases should be the clinicians involved in the follow-up of patients in the acute and chronic phases of the disease. Tests to be performed between 6 and 12 months were the same as those in the initial assessment (100% agreement), except for dorsal and lumbar spine radiographs. There was no agreement (71.4%) regarding the need to perform a bone density scan (DXA) every 2–4 years.

**Figure 2:**
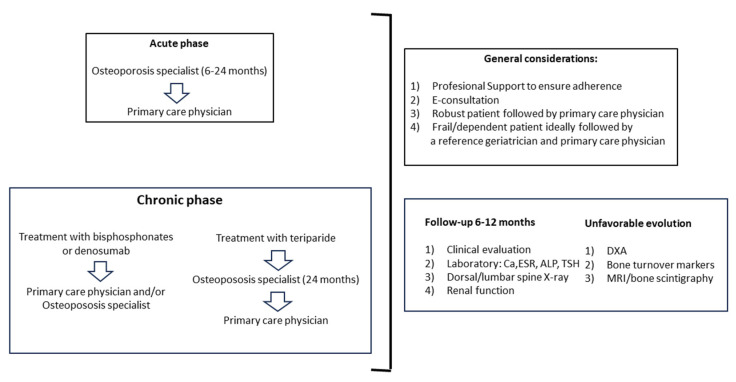
In the acute phase, patients should be followed by a specialist in osteoporosis for the initial 6–24 months and then by a primary care physician. In the chronic phase, patients treated with bisphosphonates or denosumab can be followed indistinctively by a primary care physician or a specialist in osteoporosis, whereas patients receiving teriparatide should be followed by a specialist in osteoporosis for 24 months and then by a primary care physician. General considerations and tests to be performed are summarized in the right panels. Abbreviations: E-consultation: electronic consultation; Ca: calcium; ESR: erythrocyte sedimentation rate; ALP: alkaline phosphatase; TSH: thyroid-stimulating hormone; X-ray: standard radiographs; DXA: dual-energy X-ray absorptiometry; MRI: magnetic resonance imaging.

The main recommendations to consider in the follow-up of older people with VFF are summarized in Figure 2.

## 4. Discussion

We found a relatively high consensus rate among experts of different specialties regarding the initial diagnostic tests, non-pharmacological and pharmacological treatment, and follow-up of older patients with VFF. Interesting aspects of this project include the consideration of osteoporosis from a multidisciplinary approach, the focus of the survey on VFF only, and the differentiation of older people into the three categories of “robust”, “frail” and “severely disabled”, based on their physiological vulnerability, functional and mental capacity, and family/social support [14].

The number of people aged 80 years or older is expected to triple between 2020 and 2050, to reach 426 million, and every country in the world is currently experiencing growth in both the size and the proportion of older people in its population [32]. In fact, the EU-27 is projected to be close to half a million centenarians by 2050 [33]. While increased longevity and improved health at older ages represent one of the crowning achievements of the 20th century, societal aging can affect economic growth, patterns of work and retirement, the way that families function, and the ability of governments and communities to provide adequate resources for older people, and particularly, the prevalence of chronic diseases and disability. In fact, the burden of frailty in the older population is a major public health challenge [34]. In a systematic review and meta-analysis of five studies that included a large number of community-dwelling older people (n = 3528), frailty and pre-frailty were significant predictors of a nursing home admission (odds ratio [OR] 5.85, 95% confidence interval [CI] 2.94–10.60, *p* < 0.001) [35]. Also, it has been shown that frailty is a dynamic status with transitions of improvement, worsening, or maintaining the same frailty status over time [36]. An interesting and emergent concept is osteosarcopenia: the presence of osteopenia/osteoporosis and sarcopenia. The prevalence of osteosarcopenia in community-dwelling older people ranges between 5% and 37%, with the highest rates observed in those with fragility fractures. Therefore, clinicians should be aware of screening for the presence of osteosarcopenia and treat it appropriately [37].

Fragility fractures in older people are the main clinical consequences of osteoporosis, and older patients who have suffered from a fragility fracture, named as a guide, index, or sentinel fracture, should be treated promptly to prevent the high risk of a new fracture (“imminent risk of fracture”), especially within the first 2 years after an index fracture [38]. However, recommendations for the management of osteoporosis in this age group (or in very old patients over 80 years) are not explicitly included in most clinical practice guidelines for osteoporosis. In order to provide specific recommendations for older people with VFF, all national guidelines of the main specialties involved in the care of patients with osteoporosis published in the last years (since 2014) were reviewed. The participants were experts with proven experience in the management of patients with osteoporosis from the perspective of each particular specialty.

In the section on diagnostic tests in the initial assessment of older patients with VFF, it should be noted that the evaluation of bone turnover markers and the use of the FRAX^®^ tool did not achieve consensus, whereas clinical assessment, laboratory tests with a determination of calcium and vitamin D, the erythrocyte sedimentation rate, ALP, TSH, proteinography, and renal function, as well as radiographic examination of the dorsal and lumbar spine, were usually recommended. A specific question regarding DXA was not included in the section on diagnostic methods, given that in our country, it is usually indicated to perform screening for osteoporosis using DXA at least initially in all women aged 65 years or older and in all men aged 70 years or older, which is in agreement with the recommendations by the International Society for Clinical Densitometry [39]. A routine baseline DXA may not be indicated in patients with mobility difficulties or when the diagnosis of osteoporosis can be made straightforwardly based on the history of previous fragility fractures.

Regarding the choice of questions for non-pharmacological measures, the panel agreed that these were the basic specific and general questions that are or should be used for the prevention of the risk of fractures in the general population. In relation to the questions on pharmacological treatment, those selected included the main aspects of the treatment of osteoporosis and the prevention of fractures in patients at risk. Given that the treatment of patients with fractures should be established in the long-term, we considered that it is important to favor sequential or combined treatment with a bone-forming agent first, followed by an antiresorptive drug (the initial two questions in the pharmacological treatment section in Table 2), as in the general population. Moreover, the renal function for drugs excreted by the kidneys and oral tolerance should be considered when selecting a pharmacological treatment, as shown in the following questions (3, 4, and 5 in Table 2), since these two parameters are essential to promote treatment adherence and to avoid iatrogenic and adverse events.

Concerning non-pharmacological treatment, stopping smoking and alcohol intake were recommended together with calcium/vitamin D dietary supplementation and the practice of regular physical exercise. Although the optimal physical activity programs remain unclear, there is evidence of the beneficial effects of exercise interventions on different outcomes, including balance performance, muscle strength, functional ability, body composition, and a reduction in frailty [40]. Likewise, combined calcium plus vitamin D supplementation had a significant effect on the reduction of the risk of falls (OR for the risk of suffering at least one fall, 0.87, 95% CI: 0.80–0.94) in a systematic review and meta-analysis of 26 articles in which 16,540 older people were included [41]. Therefore, it is important to stratify the risk of fracture to select the non-pharmacologic plan of choice [42].

In relation to drug therapy, the use of teriparatide for 24 months and later switching to denosumab or bisphosphonates was the only recommendation that achieved consensus. The effectiveness of teriparatide treatment in older patients to reduce the risk of fragility fractures has been demonstrated in several real-world studies [43,44,45].

The experts agreed on the importance of individualized treatment for frail and older people with severe disability, to assess the risk of falls, to evaluate muscle mass and sarcopenia in this population, and to follow the general recommendations for adults for the management of osteoporosis in robust male and female older people. Primary care physicians and/or specialists in osteoporosis should be involved in the follow-up of patients with VFF in the acute and chronic phases when treated with bisphosphonates or denosumab, whereas a geriatrician should be responsible for the follow-up of frail or disabled subjects. However, despite achieving 100% agreement on the role of primary care physicians in the management of fragility fractures in older people, it has been shown that the diagnosis and treatment of osteoporosis, as well as fracture risk assessment, is frequently lacking in the primary care setting [46,47]. Therefore, effective communication with primary care professionals (e-consultation or other resources of telemedicine) is a critical aspect of the appropriate interdisciplinary management of older patients with VFF.

Finally, we believe that the approach of this guide is really interesting and highly applicable to clinical practice since it focuses on older people (over 80 years of age) with prevalent VFF, since most guides focus on the treatment of postmenopausal osteoporosis or older men in general, with/without fracture, without focusing so much on this age range. Furthermore, we classify older people according to the three most accepted categories in current geriatrics based on their physical vulnerability, functional and mental capacity, and family/social support. Likewise, recommendations are given both for the diagnostic assessment and clinical follow-up, as well as for the non-pharmacological measures and available pharmacological treatment, with a special mention given to treatment with osteo-forming agents, usually not considered in this age range.

The limitations of this study include the reduced number of members of the task force, but a distinct feature of this is the multidisciplinary approach of the study based on the opinions of different specialists with expertise in the care of older people with osteoporosis/bone diseases. It should be noted that all panelists were professionals of recognized scientific prestige, with more than 20 years of experience, and were in charge or as coordinators of specific units of bone metabolism. However, the potential bias of the different experiences of each clinician and their own perspectives in the choice of diagnostic tests or even therapy that may be conditioned by economic issues of their institutional context cannot be ignored. From the present study, we have learned that, in order to carry out more consistent recommendations, a greater number of panelists would be desirable and that we also need to include other specialties, such as internal and emergency medicine, with at least two members per specialty plus two methodologists; around 18–20 panelists in total. Lastly, when this consensus was reached, romosozumab was not yet approved in our country, but the panelists generally agreed that the ideas applied to teriparatide could also be applied to romosozumab in a large number of cases once approved, excluding those with a history of myocardial infarction or stroke of any degree of severity.

In summary, the present recommendations are intended to facilitate the appropriate diagnosis, treatment, and follow-up of older people suffering from VFF. Experts agreed on the need for clinical assessment and laboratory tests, including the erythrocyte sedimentation rate, proteinography, calcium, vitamin D, alkaline phosphatase, and thyroid-stimulating hormone. Non-pharmacological measures include calcium/vitamin D supplementation, withdrawal of toxic habits, and personalized physical exercise. In patients at high risk of fracture, teriparatide for 24 months and then switching to denosumab or bisphosphonates is recommended. Prevention of new falls is just as important as the treatment of vertebral fractures. Geriatricians have a relevant role in the care of these patients. Furthermore, a more collaborative care model between different levels of health care is a key component for the early detection and prevention of fragility fractures in older people.

## Figures and Tables

**Table 1:** Recommended diagnostic tests for the evaluation and management of osteoporosis in older people with vertebral fragility fracture.

Item	Agreement %	9-Point Likert Scale Mean (SD)
Anamnesis and clinical assessment	**100**	9 (0)
Laboratory tests, including levels of calcium (Ca), vitamin D, erythrocyte sedimentation rate, alkaline phosphatase (ALP), thyroid-stimulating hormone (TSH), and proteinography	**100**	9 (0)
Dorsal and lumbar spine radiographs	**85.7**	8.4 (1.5)
Measurement of renal function	**100**	8.7 (0.8)
Specific laboratory tests: immunofixation and parathyroid hormone (PTH) levels	57.1	6.7 (2.7)
Bone turnover markers (C-terminal telopeptide of type I collagen (CTX) and procollagen type 1 N propeptide (P1NP))	28.6	5.1 (3.1)
Magnetic resonance imaging (MRI)/bone scintigraphy	57.1	6.3 (3.1)
Additional question FRAX^®^ (major osteoporotic fracture (MOF): threshold ≥ 10% without bone mineral density (BMD) or ≥7.5% with BMD or >3% for the hip)	28.6	6 (2.7)

SD: standard deviation; consensus in favor in **bold**. FRAX^®^: Fracture Risk Assessment Tool.

**Table 2:** Recommended non-pharmacological actions and pharmacological treatment in older people with vertebral fragility fracture.

Item	Agreement %	9-Point Likert Scale Mean (SD)
*Non-pharmacological actions*		
Administration of calcium/vitamin D (dietary or supplementation)	**100**	8.9 (0.4)
Breaking toxic habits (tobacco and alcohol)	**100**	9 (0)
Personalized physical exercise	**85.7**	8.7 (0.8)
Orthosis treatment if needed after individual assessment and especially in case of acute vertebral fracture	57.1	7.3 (2.7)
Reduction in caffeine consumption	0	4.4 (1.9)
Pharmacological treatment *		
− Administration of teriparatide for 24 months and then switch to denosumab or to an oral (risedronate or alendronate) or intravenous (zoledronate) bisphosphonate	**85.7**	9 (0)
− Administration of combined therapy (teriparatide + denosumab) only in individualized cases of severe osteoporosis (the current evidence does not support the use of combined therapy in the general management of osteoporosis, but only in individualized cases of severe osteoporosis)	42.9	6.5 (1.9)
− In patients with good renal function and oral tolerance, bisphosphonates would be the recommended treatment	71.4	8 (2.0)
− In patients with good renal function and poor oral tolerance, denosumab or zoledronate would be the recommended treatment	71.4	8.2 (2.0)
− In patients with poor renal function and oral tolerance, denosumab would be the recommended treatment	71.4	8.7 (0.8)

SD: standard deviation; consensus in favor in **bold**. * These recommendations include patients who had presented at least one previous VFF; that is, high-risk patients, with many of them at very high risk or even at imminent risk of a new fracture if the index VFF had occurred in the previous 12–24 months.

**Table 3:** Recommended actions at follow-up of older people with vertebral fragility fracture.

Item	Agreement %	9-Point Likert Scale Mean (SD)
*Classification of older people*		
− Robust patient: treatment and follow-up according to general recommendations for osteoporosis in adult women or men	**85.7**	8.1 (1.9)
− Frail patient: assessment of the risk of falls (e.g., osteoporosis school) and evaluation of the muscle mass and sarcopenia	**100**	9 (0)
− Dependent patient with severe disability: treatment individualized to the degree of dependence and emphasis on non-pharmacological actions	**85.7**	8.7 (0.8)
*Follow-up schedule*		
− In the acute phase, the patient should be attended by a specialist in osteoporosis (6–24 months according to the clinical course and the prescribed treatment) and then by a primary care physician (through e-consultation support)	**100**	8.9 (0.4)
− In the chronic phase, if the patient is treated with bisphosphonates, they should be attended by the primary care physician and/or a specialist in osteoporosis	**100**	9 (0)
− In the chronic phase, if the patient is treated with denosumab, they should be attended by the primary care physician and/or a specialist in osteoporosis	**100**	9 (0)
− In the chronic phase, if the patient is treated with teriparatide, they should be attended first by a specialist in osteoporosis (24 months) and then by the primary care physician	**100**	9 (0)
− Sought professional support (primary care physician or other specialists) to ensure adherence to treatment	**100**	8.9 (0.4)
− E-consultation support at follow-up	**100**	8.9 (0.4)
− In the case of a robust patient: follow-up by a primary care physician	**100**	9 (0)
− In the case of frail or dependent patients (severe disability), follow-up mainly by a geriatrician	**85.7**	8.6 (1.1)
*Tests to be performed between 6 and 12 months*		
− Anamnesis and clinical assessment	**100**	9 (0)
− Laboratory tests, including levels of calcium (Ca), vitamin D, erythrocyte sedimentation rate, alkaline phosphatase (ALP), and thyroid-stimulating hormone (TSH)	**100**	9 (0)
− Dorsal and lumbar spine radiographs	71.4	8.1 (1.6)
− Measurement of renal function	**100**	9 (0)
*Tests to be performed when the clinical course is unfavorable*		
− Dual X-ray absorptiometry (DXA) every 2–4 years *	71.4	7.9 (1.5)
− Bone turnover markers (C-terminal telopeptide of type I collagen (CTX), procollagen type 1 N propeptide (P1NP))	42.9	6.1 (2.7)
− Magnetic resonance imaging (MRI)/bone scintigraphy	57.1	6.9 (2.7)

SD: standard deviation; consensus in favor in **bold**. * DXA at intervals of 2–4 years can be performed independently of the clinical course.

## Data Availability

Study data are available from the corresponding author upon request.

## Supplementary Materials

The following supporting information can be downloaded at: https://www.mdpi.com/article/10.3390/geriatrics9020024/s1, Table S1: Details of the study questionnaire. Reference [48] is cited in the Supplementary Materials.

## References

[1] Kanis J.A., Melton L.J., Christiansen C., Johnston C.C., Khaltaev N. (1994). The diagnosis of osteoporosis. J. Bone Miner. Res..

[2] Castañeda S., Costa R. (2021). Clinical profile of osteoporosis in Spain. Rev. Clin. Esp..

[3] Somma T., De Rosa A., Mastantuoni C., Esposito F., Meglio V., Romano F., Ricciardi L., de Divitiis O., Disomma C. (2022). Multidisciplinary management of osteoporotic vertebral fractures. Minerva Endocrinol..

[4] Fried L.P., Tangen C.M., Walston J., Newman A.B., Hirsch C., Gottdiener J., Seeman T., Tracy R., Kop W.J., Burke G. (2001). Cardiovascular Health Study Collaborative Research Group. Frailty in older adults: Evidence for a phenotype. J. Gerontol. A Biol. Sci. Med. Sci..

[5] Ong T., Bin Syed Ali S.A., Sahota O. (2021). The presentation of older people with vertebral fragility fractures to a university hospital: A cross-sectional analysis. Curr. Rheumatol. Rev..

[6] Stanghelle B., Bentzen H., Giangregorio L., Pripp A.H., Skelton D.A., Bergland A. (2020). Effects of a resistance and balance exercise programme on physical fitness, health-related quality of life and fear of falling in older women with osteoporosis and vertebral fracture: A randomized controlled trial. Osteoporos. Int..

[7] Ong T., Kantachuvesiri P., Sahota O., Gladman J.R.F. (2018). Characteristics and outcomes of hospitalised patients with vertebral fragility fractures: A systematic review. Age Ageing.

[8] Kutsal F.Y., Ergin Ergani G.O. (2021). Vertebral compression fractures: Still an unpredictable aspect of osteoporosis. Turk. J. Med. Sci..

[9] Suzuki N., Ogikubo O., Hansson T. (2008). The course of the acute vertebral body fragility fracture: Its effect on pain, disability and quality of life during 12 months. Eur. Spine J..

[10] Sale J.E., Beaton D., Posen J., Elliot-Gibson V., Bogoch E. (2011). Systematic review on interventions to improve osteoporosis investigation and treatment in fragility fracture patients. Osteoporos. Int..

[11] Bukata S.V., DiGiovanni B.F., Friedman S.M., Hoyen H., Kates A., Kates S.L., Mears S.C., Mendelson D.A., Serna F.H., Sieber F.E. (2011). A guide to improving the care of patients with fragility fractures. Geriatr. Orthop. Surg. Rehabil..

[12] Orimo H., Ito H., Suzuki T., Araki A., Hosoi T., Sawabe M. (2006). Reviewing the definition of “elderly”. Geriatr. Gerontol. Int..

[13] Santamaría-Peláez M., González-Bernal J., González-Santos J., Soto-Cámara R. (2021). Differences between robust, frail, prefrail and dependent institutionalized older people. Aten. Primaria.

[14] Woolford S.J., Sohan O., Dennison E.M., Cooper C., Patel H.P. (2020). Approaches to the diagnosis and prevention of frailty. Aging Clin. Exp. Res..

[15] Ritt M., Ritt J.I., Sieber C.C., Gaßmann K.G. (2017). Comparing the predictive accuracy of frailty, comorbidity, and disability for mortality: A 1-year follow-up in patients hospitalized in geriatric wards. Clin. Interv. Aging.

[16] Rossini M., Adami S., Bertoldo F., Diacinti D., Gatti D., Giannini S. (2016). Guidelines for the diagnosis, prevention and management of osteoporosis. Reumatismo.

[17] Kanis J.A., Cooper C., Rizzoli R., Reginster J.Y., Scientific Advisory Board of the European Society for Clinical and Economic Aspects of Osteoporosis (ESCEO) and the Committees of Scientific Advisors and National Societies of the International Osteoporosis Foundation (IOF) (2019). European guidance for the diagnosis and management of osteoporosis in postmenopausal women. Osteoporos. Int..

[18] Gregson C.L., Armstrong D.J., Bowden J., Cooper C., Edwards J., Gittoes N.J.L., Harvey N., Kanis J., Leyland S., Low R. (2022). UK clinical guideline for the prevention and treatment of osteoporosis. Arch. Osteoporos..

[19] Castañeda S., Moro-Álvarez M.J. (2022). Update of the osteoporosis guidelines of SEIOMM. Rev. Clin. Esp..

[20] Briot K., Roux C., Thomas T., Blain H., Buchon D., Chapurlat R. (2018). 2018 update of French recommendations on the management of postmenopausal osteoporosis. Jt. Bone Spine.

[21] Reid I.R. (2021). Revisiting osteoporosis guidelines. Lancet Diabetes Endocrinol..

[22] Buckley L., Guyatt G., Fink H.A., Cannon M., Grossman J., Hansen K.E. (2017). American College of Rheumatology guideline for the prevention and treatment of glucocorticoid-induced osteoporosis. Arthritis Rheumatol..

[23] Wang M., Bolland M., Grey A. (2016). Management recommendations for osteoporosis in clinical guidelines. Clin. Endocrinol..

[24] Harvey N.C., McCloskey E., Kanis J.A., Compston J., Cooper C. (2018). Cost-effective but clinically inappropriate: New NICE intervention thresholds in osteoporosis (Technology Appraisal 464). Osteoporos. Int..

[25] Guía Clínica SEIOMM 2014. https://seiomm.org/guia_clinicas/guia-clinica-seiomm-2014/.

[26] SEMG 2015 Sociedad Española de Médicos Generales y de Familia. https://www.semg.es/.

[27] Etxebarria-Foronda E., Caeiro-Rey J.R., Larrainzar-Garijo R., Vaquero-Cervino E., Roca-Ruiz L., Mesa-Ramo M., Perez J.M., Carpintero-Benitez P., Cebrian A.F., Gil-Garay E. (2015). SECOT-GEIOS guidelines in osteoporosis and fragility fracture. An update. Rev. Esp. Cir. Ortop. Traumatol..

[28] semFYC Sociedad Española de Medicina Familiar y Comunitaria Grupo de Trabajo de Enfermedades Reumatológicas de la semFYC. Osteoporosis. Management: Prevention, Diagnosis and Treatment. https://www.semfyc.es/wp-content/uploads/2016/03/Libro_Osteoporosis14_Def.pdf.

[29] Naranjo Hernández A., Díaz del Campo Fontecha P., Aguado Acín M.P., Arboleya Rodríguez L., Casado Burgos E., Castañeda S., Areste J.F., Gifre L., Vaquero C.G., Rodriguez G.C. (2019). Recommendations by the Spanish Society of Rheumatology on Osteoporosis. Reumatol. Clin..

[30] Servicio Madrileño de Salud (2015). Recomendaciones Para la Valoración y Tratamiento de la Osteoporosis Primaria en Mujeres de la Comunidad de Madrid.

[31] Varsavskya M., Romero Muñoz M., Ávila Rubio V., Becerra A., García Martín A., Martínez Díaz-Guerra G. (2018). Consensus documento on osteoporosis in males. Endocrinol. Diabetes Nutr..

[32] World Health Organization Ageing and Health. https://www.who.int/news-room/fact-sheets/detail/ageing-and-health.

[33] Ageing Europe—Statistics on Population Developments. https://ec.europa.eu/eurostat/statistics-explained/index.php?title=Ageing_Europe_-_statistics_on_population_developments.

[34] Buckinx F., Rolland Y., Reginster J.Y., Ricour C., Petermans J., Bruyère O. (2015). Burden of frailty in the elderly population: Perspectives for a public health challenge. Arch. Public Health.

[35] Kojima G. (2019). Frailty as a predictor of nursing home placement among community-dwelling older adults: A systematic review and meta-analysis. J. Geriatr. Phys. Ther..

[36] Kojima G., Taniguchi Y., Iliffe S., Jivraj S., Walters K. (2019). Transitions between frailty states among community-dwelling older people: A systematic review and meta-analysis. Ageing Res. Rev..

[37] Kirk B., Zanker J., Duque G. (2020). Osteosarcopenia: Epidemiology, diagnosis, and treatment-facts and numbers. J. Cachexia Sarcopenia Muscle.

[38] Wong R.M.Y., Wong P.Y., Liu C., Wong H.W., Chung Y.L., Chow S.K.H., Law S.W., Cheung W.H. (2022). The imminent risk of a fracture-existing worldwide data: A systematic review and meta-analysis. Osteoporos. Int..

[39] Krueger D., Tanner S.B., Szalat A., Malabanan A., Prout T., Lau A., Rosen H.N., Shuhart C. (2023). DXA Reporting Updates: 2023 Official Positions of the International Society for Clinical Densitometry. J. Clin. Densitom..

[40] De Labra C., Guimaraes-Pinheiro C., Maseda A., Lorenzo T., Millán-Calenti J.C. (2015). Effects of physical exercise interventions in frail older adults: A systematic review of randomized controlled trials. BMC Geriatr..

[41] Wu H., Pang Q. (2017). The effect of vitamin D and calcium supplementation on falls in older adults: A systematic review and meta-analysis. Orthopade.

[42] Kanis J.A., Harvey N.C., McCloskey E., Bruyère O., Veronese N., Lorentzon M., Cooper C., Rizzoli R., Adib G., Al-Daghri N. (2020). Algorithm for the management of patients at low, high and very high risk of osteoporotic fractures. Osteoporos. Int..

[43] Gallagher J.C., Genant H.K., Crans G.G., Vargas S.J., Krege J.H. (2005). Teriparatide reduces the fracture risk associated with increasing number and severity of osteoporotic fractures. J. Clin. Endocrinol. Metab..

[44] Burge R.T., Disch D.P., Gelwicks S., Zhang X., Krege J.H. (2017). Hip and other fragility fracture incidence in real-world teriparatide-treated patients in the United States. Osteoporos. Int..

[45] Chen C.H., Elsalmawy A.H., Ish-Shalom S., Lim S.J., AlAli N.S., Cunha-Borges J.L. (2022). The effect of teriparatide treatment on the risk of fragility fractures in postmenopausal women with osteoporosis: Results from the Asian and Latin America Fracture Observational Study (ALAFOS). Calcif. Tissue Int..

[46] Bell A., Kendler D.L., Khan A.A., Shapiro C.M., Morisset A., Leung J.P. (2022). A retrospective observational study of osteoporosis management after a fragility fracture in primary care. Arch. Osteoporos..

[47] Martínez-Laguna D., Carbonell C., Bastida J.C., González M., Micó-Pérez R.M., Vargas F. (2022). Prevalence and treatment of fragility fractures in Spanish primary care: PREFRAOS study. Arch. Osteoporos..

[48] Riancho J.A., Peris P., González-Macías J., Pérez-Castrillón J.L., SEIOMM Osteoporosis Guidelines Writing Group (2022). Executive summary clinical practice guideline of postmenopausal, glucocorticoid-induced and male osteoporosis (2022 update). Spanish Society for Bone and Mineral Metabolism Investigation (SEIOMM). Rev. Clin. Esp..

