# Supplementary material for: Management of Vertebral Fragility Fracture in Older People: Recommendations from a Spanish Consensus of Experts
Source: Geriatrics (Basel). 2024 Feb 23;9(2):24. doi: 10.3390/geriatrics9020024 (PMC10961758; doi:10.3390/geriatrics9020024)
Supplement: Supplementary file 1 [file geriatrics-09-00024-s001.zip › geriatrics-2722100-supplementary.pdf]

Study questionnaire

Older people with vertebral fragility fracture

A. DIAGNOSTIC TESTS

Please, indicate your degree of agreement with each of the following statements regarding the diagnostic tests recommended to be performed in older people with vertebral fragility fracture

	1	2	3	4	5	6	7	8	9
1. Anamnesis and clinical assessment									
2. Laboratory tests including levels of calcium (Ca), vitamin D, erythrocyte sedimentation rate, alkaline phosphatase (ALP), thyroid-stimulating hormone (TSH), and protein electrophoresis									
3. Dorsal and lumbar spine radiographs									
4. Measurement of renal function									

Remember: 1: Strongly disagree and 9: Strongly agree

	1	2	3	4	5	6	7	8	9
5. Laboratory tests: immunofixation, parathyroid hormone (PTH)									
6. Bone turnover markers (C-terminal telopeptide of type I collagen [CTX], procollagen type 1 N propeptide [P1NP])									
7. Magnetic resonance imaging (MRI)/bone scintigraphy									

Remember: 1: Strongly disagree and 9: Strongly agree

Additional question

Please, indicate your degree of agreement for the use of FRAX® tool to assess the risk of fracture in older people with vertebral fragility fracture

	1	2	3	4	5	6	7	8	9
FRAX® (major osteoporotic fracture [MOF]: threshold ≥ 10% without bone mineral density [BMD] or ≥ 7.5% with BMD or > 3% for the hip)									

Remember: 1: Strongly disagree and 9: Strongly agree

Please, indicate if you have any comment regarding the aforementioned questions:

B. NON-PHARMACOLOGICAL TREATMENT OF OLDER PEOPLE

Please, indicate your degree of agreement with each of the following statements regarding the treatment recommended for older people with vertebral fragility fracture

	1	2	3	4	5	6	7	8	9
8. Administration of calcium/vitamin D (dietary or supplementation)									
9. Breaking toxic habits (tobacco and alcohol)									
10. Personalized physical exercise									

Remember: 1: Strongly disagree and 9: Strongly agree

	1	2	3	4	5	6	7	8	9
11. Orthosis treatment if needed after individual assessment and especially in case of acute vertebral fracture									
12. Reduction of caffeine consumption									

Remember: 1: Strongly disagree and 9: Strongly agree

Please, indicate if you have any comment regarding the aforementioned questions:

C. PHARMACOLOGICAL TREATMENT OF OLDER PEOPLE

Please, indicate your degree of agreement with each of the following statements regarding the pharmacological treatment recommended for older people at high risk (≥ 2 vertebral fractures) of vertebral fragility fracture:

Risk is currently categorized as 'high risk' and 'very high risk'. Imminent risk may be included in the high risk category (but not in the very high risk) according to the recent guideline of the Spanish Society for Bone Research and Mineral Metabolism (SEIOMM) 2022 [48]

	1	2	3	4	5	6	7	8	9
13. Administration of teriparatide for 24 months and then switch to denosumab or to an oral (risedronate and alendronate) or intravenous (zoledronate) bisphosphonate									
14. Administration of combined therapy (teriparatide + denosumab) only in individualized cases of severe osteoporosis (the current evidence does not support the use of combined therapy in the general management of osteoporosis, but only in individualized cases of severe osteoporosis)									

Remember: 1: Strongly disagree and 9: Strongly agree

	1	2	3	4	5	6	7	8	9
15. In patients with good renal function and oral tolerance, bisphosphonates would be the recommended treatment									
16. In patients with good renal function and poor oral tolerance, denosumab or zoledronate would be the recommended treatment									
17. In patients with poor renal function and oral tolerance, denosumab would be the recommended treatment									

Remember: 1: Strongly disagree and 9: Strongly agree

Please, indicate if you have any comment regarding the aforementioned questions:

D. FOLLOW-UP OF OLDER PATIENTS

Regarding the classification of an older people into 'robust', 'frail' and 'dependent', please indicate your degree of agreement with each of the following statements:

	1	2	3	4	5	6	7	8	9
18. Robust patient: treatment and follow-up according to general recommendations for osteoporosis in women or men									
19. Frail patient: assessment of the risk of falls (e.g. osteoporosis school) and evaluation of the muscle mass and sarcopenia									
20. Dependent patient: treatment individualized to the degree of dependence and emphasis on non-pharmacological measures									

Remember: 1: Strongly disagree and 9: Strongly agree

Please, indicate your degree of agreement with each of the following statements regarding the follow-up of older people with vertebral fragility fracture:

	1	2	3	4	5	6	7	8	9
21. In the acute phase, the patient should be attended by the specialist in osteoporosis (6-24 months according to the clinical course and the prescribed treatment) and then by the primary care physician (with e-consultation support)									
22. In the chronic phase, if the patient is treated with bisphosphonates should be attended by the primary care physician and/or the specialist in osteoporosis									
23. In the chronic phase, if the patient is treated with denosumab should be attended by the primary care physician and/or the specialist in osteoporosis									
24. In the chronic phase, if the patient is treated with teriparatide should be attended first by the specialist in osteoporosis (24 months) and then by the primary care physician									
25. Sought professional support (primary care physician or other specialists) to ensure adherence to treatment									
26. E-consultation support at follow-up									
27. In case of robust patient: follow-up by a primary care physician									
28. In case of frail or dependent patient: follow-up mainly by a geriatrician									

Remember: 1: Strongly disagree and 9: Strongly agree

Please, indicate your degree of agreement with each of the following statements regarding the recommended tests to be performed in older people with vertebral fragility fracture between 6 and 12 months:

	1	2	3	4	5	6	7	8	9
29. Anamnesis and clinical assessment									
30. Laboratory tests including levels of calcium (Ca), vitamin D, erythrocyte sedimentation rate, alkaline phosphatase (ALP), and thyroid-stimulating hormone (TSH)									
31. Dorsal and lumbar spine radiographs									
32. Measurement of renal function									

Remember: 1: Strongly disagree and 9: Strongly agree

Please, indicate your degree of agreement with each of the following statements regarding optional tests to be performed in older people with vertebral fragility fracture if the clinical course is unfavorable:

	1	2	3	4	5	6	7	8	9
33. Bone density scan (DEXA) every 2-4 years									
34. Bone turnover markers (C-terminal telopeptide of type I collagen [CTX], procollagen type 1 N propeptide [P1NP])									
35. Magnetic resonance imaging (MRI)/bone scintigraphy									

Remember: 1: Strongly disagree and 9: Strongly agree

Please, indicate if you have any comment regarding the aforementioned questions:
